# Comparative Study on Microstructural Characteristics and Fatigue Performance of 10 mm Thick 5083-H111 Aluminum Alloy Plates Processed by Different Cutting Methods

**DOI:** 10.3390/ma19214656

**Published:** 2026-11-01

**Authors:** Xin Li, Qiang Guo, Wanguo Li, Meijie Jia, Lei Wang

**Affiliations:** 1 Key Laboratory of Automobile Materials, School of Materials Science and Engineering, Jilin University, Changchun 130022, China; 2 CRRC Changchun Railway Vehicles Co., Ltd., Changchun 130062, China

**Keywords:** 5083-H111 aluminum alloy, laser cutting, fatigue performance, fatigue risk factor, fatigue failure mechanism

## Abstract

This study evaluates the applicability of laser cutting to 10 mm thick 5083-H111 aluminum alloy plates by comparing fiber laser cutting, waterjet cutting, and mechanical grinding. The cut-edge morphology, microstructure, tensile properties, and fatigue behavior were systematically investigated, with particular emphasis on the effects of laser-induced thermal input on the heat-affected zone (HAZ), grain orientation, precipitates, and residual stress. A fatigue risk factor (*R_f_*) was further introduced to quantify fatigue cracking susceptibility. At 1 × 10^7^ cycles, the fatigue strengths of the mechanically ground specimen and the waterjet-cut, laser-cut without dross removal, and laser-cut with dross removal specimens were 119.18, 99.21, 60.19, and 67.64 MPa, respectively. Dross removal increased the fatigue strength of the laser-cut specimens by 12.38% by reducing local stress concentration, although their fatigue performance remained inferior to that of the other two processes. Periodic surface striations and heat-affected-zone degradation were identified as the primary causes of fatigue strength reduction. Fatigue cracks mainly initiated at dross roots, surface striations, and nearby defects in the laser-cut specimens. The laser-cut specimens exhibited the highest fatigue risk factor (*R_f_* = 1.32), compared with 0.36 for waterjet cutting and 0.16 for mechanical grinding, corresponding to the lowest fatigue life.

## 1. Introduction

Rail transit equipment is increasingly developing toward lightweight and high-speed operation. Owing to their low density, high specific strength, and excellent corrosion resistance, aluminum alloys have been widely used in load-bearing structures of rail vehicle bodies [1,2,3,4,5]. With the continuously increasing requirements for manufacturing quality, the cutting quality of 10 mm thick 5083-H111 aluminum alloy plates commonly used in rail transit directly affects the accuracy of subsequent assembly and welding, and is closely associated with structural safety and service life. Therefore, the rational selection and optimization of cutting processes are of considerable engineering significance [6,7].

Conventional cutting methods for medium-thick aluminum alloy plates exhibit distinct advantages and limitations. mechanical Mechanical grinding can provide high dimensional accuracy and good surface quality, although it is relatively costly and involves substantial material removal [8,9]. Abrasive waterjet cutting is a cold-processing method with limited thermal damage and has been widely investigated for difficult-to-machine and aluminum alloys [10,11]. Laser cutting offers high processing flexibility and efficiency, but the resulting surface quality is strongly affected by the cutting parameters and surface-roughness characteristics [12,13]. Hybrid waterjet–laser processing has also been explored to improve cutting quality and reduce thermal effects [14]. Wire electrical discharge machining is suitable for the precision processing of electrically conductive materials, but its relatively low machining efficiency restricts its application in mass production. At present, waterjet cutting is commonly used to process 10 mm thick 5083-H111 aluminum alloy plates for rail transit applications. As a cold-cutting process, waterjet cutting minimizes thermal damage; however, its relatively low processing efficiency, high abrasive consumption, and substantial equipment maintenance requirements lead to increased operating costs. Furthermore, the treatment of solid waste and wastewater poses additional environmental challenges. Consequently, existing cutting technologies face difficulties in simultaneously meeting the requirements for high processing efficiency, flexibility, dimensional accuracy, and environmental sustainability, highlighting the need to explore alternative cutting processes with improved overall performance.

Laser cutting offers several advantages, including non-contact processing, high cutting speed, high precision, excellent forming flexibility, and ease of integration with automated manufacturing systems, and has been widely applied to thin-sheet processing [15,16,17]. With the rapid development of high-power fiber laser technology, its potential for processing medium-thick aluminum alloy plates has become increasingly evident. Nevertheless, engineering applications still face challenges related to cut quality and in-service performance. During laser cutting, a high-energy-density heat source locally melts the material, while an assist gas removes the molten metal from the kerf. This thermal process inevitably generates a heat-affected zone (HAZ) along the cut edge, which may alter grain morphology, precipitate characteristics, and elemental distribution. These microstructural changes can further modify microhardness and residual stress distributions, thereby affecting the static mechanical properties of the component [18,19,20,21,22,23]. For critical rail vehicle components subjected to cyclic loading, cut-edge defects, thermally induced microstructural degradation, and residual stresses may act synergistically to promote fatigue crack initiation and reduce long-term structural reliability. Therefore, the microstructure and mechanical performance of laser-cut 10 mm thick 5083-H111 aluminum alloy components need to be systematically evaluated and compared with those produced by conventional cutting processes.

Current studies on laser cutting of aluminum alloys for rail transit applications still present two major limitations. First, a stable laser-cutting process window for 10 mm thick 5083-H111 aluminum alloy has not yet been well established. Process parameters are often determined empirically, which can readily result in defects such as kerf taper, high surface roughness, and bottom-edge dross, making it difficult to achieve both high processing efficiency and satisfactory cut quality. Second, the coupled effects of laser-induced thermal input on microstructure, residual stress, and mechanical properties have not been systematically characterized through both qualitative and quantitative analyses. Evaluation based solely on the macroscopic quality of the cut edge is therefore insufficient to accurately assess the service reliability of laser-cut components. A comprehensive investigation integrating microstructural characterization, static and fatigue properties, and comparisons among different cutting processes is consequently required.

Thick 5083-H111 aluminum alloy plates used in rail transit are commonly processed by waterjet cutting. However, this method is characterized by relatively low processing efficiency, high consumable and maintenance costs, and additional challenges associated with solid-waste and wastewater treatment, making it difficult to simultaneously satisfy the requirements of high efficiency, processing flexibility, dimensional accuracy, and green manufacturing. Laser cutting offers several advantages, including non-contact processing, high cutting speed, high precision, strong processing flexibility, and easy integration with automated manufacturing systems, and has been widely applied to the processing of thin aluminum alloy sheets. With the rapid development of high-power fiber laser technology, laser cutting has shown considerable potential for the processing of medium-thick aluminum alloy plates. Nevertheless, the effects of laser processing on the cut-surface quality and in-service performance of medium-thick aluminum alloy components remain insufficiently understood. Previous studies on aluminum-alloy cutting have mainly focused on process-parameter optimization and cut quality, including kerf geometry, surface roughness, dross formation, and heat-affected-zone characteristics. However, the relationships among cutting-induced surface integrity, near-edge microstructural evolution, residual stress, and high-cycle fatigue behavior remain insufficiently understood, particularly for medium-thick 5083-H111 aluminum alloy plates used in rail transit. In contrast to studies focusing on a single cutting process or individual surface characteristics, the present work directly compares laser cutting, abrasive waterjet cutting, and mechanical grinding under the same material conditions. Surface morphology, microstructure, phase characteristics, residual stress, tensile properties, S–N behavior, and fatigue fracture characteristics are systematically correlated. Furthermore, the fatigue risk factor is introduced to quantitatively evaluate the combined influence of surface condition, residual stress, and mechanical properties on fatigue-crack susceptibility. This integrated analysis provides a clearer understanding of the mechanisms responsible for the reduced fatigue resistance of laser-cut edges and identifies practical directions for improving the fatigue performance of laser-cut 5083-H111 aluminum alloy components.

In this study, the effects of waterjet cutting and laser cutting on the surface morphology, mechanical properties, and service performance of aluminum alloy specimens were comparatively investigated, and the underlying mechanisms were analyzed. The results provide a theoretical basis for evaluating the feasibility of applying laser cutting to the engineering processing of thick aluminum alloy plates.

Furthermore, the present study focuses on a 5xxx-series aluminum alloy. Caution should therefore be exercised when extending the present findings to 6xxx-series aluminum alloys, which are heat-treatable and may exhibit greater sensitivity to the thermal effects induced by laser cutting. Consequently, the laser-cutting parameters optimized for 5083-H111 aluminum alloy cannot be directly transferred to 6xxx-series aluminum alloys without further validation.

## 2. Materials and Methods

**Figure 1:**
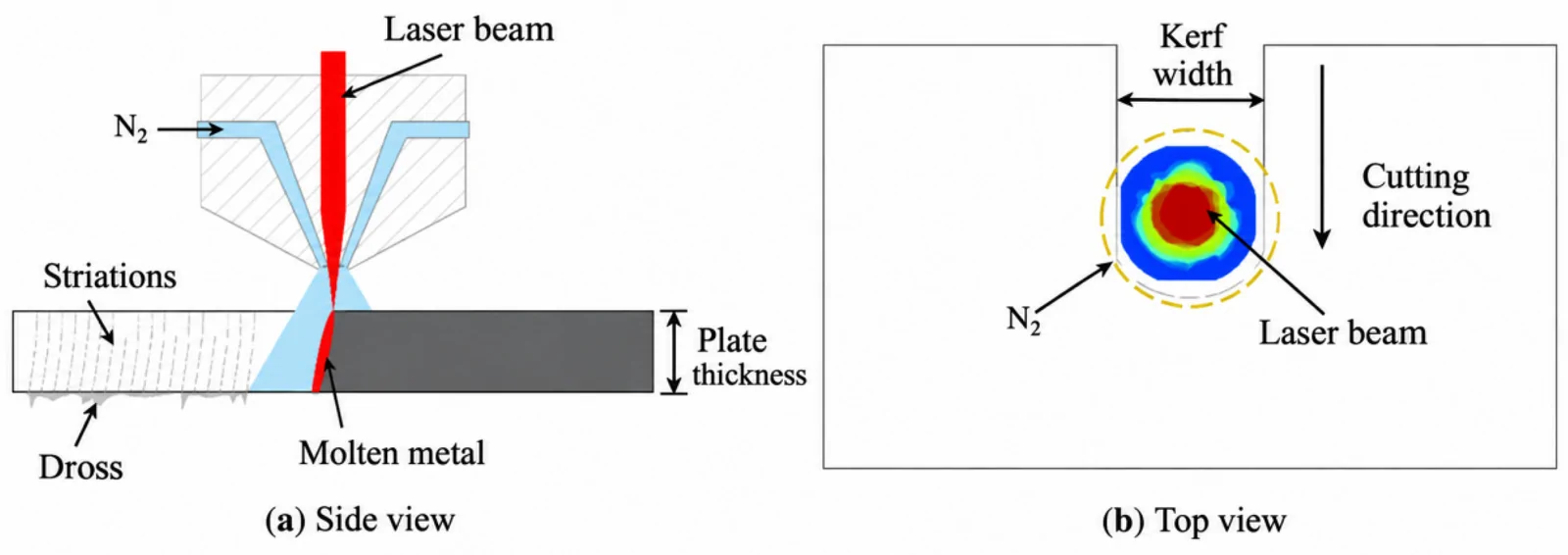
Schematic of laser cutting.

The experimental material was a 10 mm thick 5083-H111 aluminum alloy plate conforming to GB/T 3880.2-2024 [24], and its chemical composition and physical properties are listed in Table 1 and Table 2, respectively. The plate was clamped on the laser cutting machine, and the process parameters were set according to the experimental design, while the remaining parameters were kept at the default settings of the equipment. The specimen dimensions were 70 mm × 70 mm. The cutting direction was parallel to the rolling direction (RD), while the horizontal direction corresponded to the transverse direction (TD) and the thickness direction to the normal direction (ND). The total cutting length was 320 mm, and the spacing between adjacent specimens was 30 mm. The cutting layout is illustrated in Figure 1.

Laser cutting was performed using a πIIF-4020 gantry-type laser cutting machine (Suzhou Lingchuang Laser Technology Co., Ltd., Suzhou, China) equipped with a Raytools Jafiber 6 kW cutting head. The collimating and focusing focal lengths were 100 and 150 mm, respectively, and a 4.0 mm nozzle was used. The optimized parameters were 5500 W laser power, 600 mm/min cutting speed, and 2 MPa nitrogen assist-gas pressure. The laser-cutting parameters used in the subsequent comparative experiments were determined through preliminary single-factor tests, an L9 (3^3^) orthogonal design, and response surface optimization. Laser power, cutting speed, and nitrogen assist-gas pressure were selected as the main process variables, while kerf inclination, surface roughness, dross height, and the width of the microstructurally affected region were used as quality responses. The optimized parameter combination was a laser power of 5500 W, a cutting speed of 600 mm/min, and a nitrogen pressure of 2.0 MPa. Under these conditions, the predicted values of kerf inclination, surface roughness, dross height, and microstructurally affected-region width were 0.87°, 27.44 μm, 202 μm, and 82 μm, respectively, while the corresponding experimental values were 0.89°, 28.73 μm, 207 μm, and 86 μm. The relative errors were all below 5%, confirming the reliability of the selected processing conditions.

The initial specimens measured 70 mm × 70 mm × 10 mm, with the cutting direction parallel to the rolling direction. Abrasive waterjet cutting was conducted using an X–Y CNC waterjet system at 620 MPa, with a cutting accuracy of approximately ±0.1 mm. Mechanically ground reference specimens were prepared by first cutting the aluminum alloy plates to size using waterjet cutting and then grinding and polishing the cut surfaces to remove the near-surface plastic deformation and surface textures induced by waterjet erosion, thereby minimizing differences in surface condition introduced during the initial cutting operation. The material removal during grinding was on the order of several tens of micrometers, and grinding was terminated when the characteristic waterjet-cutting striations had completely disappeared. This procedure was intended to remove the surface damage introduced by waterjet cutting while minimizing additional disturbance to the near-surface microstructure caused by mechanical finishing. The mechanically ground specimens were subsequently compared with the as-waterjet-cut and laser-cut specimens to clarify the effects of the cutting processes on the properties of the aluminum alloy.

Surface roughness was characterized in the selected lower cut-edge region, where the laser-cut surface exhibited the most pronounced roughness and inclined striations. For each specimen condition, roughness was measured on three independent specimens. The reported Ra values correspond to the surface profiles acquired in this comparison region.

Metallographic specimens were extracted from the kerf-edge region by wire electrical discharge machining (WEDM) to dimensions of 6 mm × 4 mm × 10 mm. After mounting, the specimens were sequentially ground using 600#–2000# abrasive papers and polished to a mirror finish using a 2.5 μm diamond polishing agent. The polished specimens were etched with Keller’s reagent for 25 s, rinsed with alcohol, and then air-dried prior to optical and SEM observations. For optical microscopy and SEM characterization, three replicate specimens were prepared for each cutting method. The microstructural features of specimens within the same group were similar, indicating that the observed morphology was representative of the corresponding cutting process. Therefore, representative regions were selected for analysis.

For EBSD characterization, the specimens were prepared following the same mechanical grinding procedure and were subsequently electropolished using a perchloric acid–alcohol solution with a platinum electrode. Electropolishing was performed at 20 V for 16 s, while the electrolyte temperature was maintained at 3–5 °C using an ice bath. After electropolishing, the specimens were rinsed with alcohol and air-dried. EBSD characterization was conducted using a JSM-7900F field-emission scanning electron microscope (JEOL Ltd., Tokyo, Japan) equipped with an EBSD detector.

**Figure 2:**
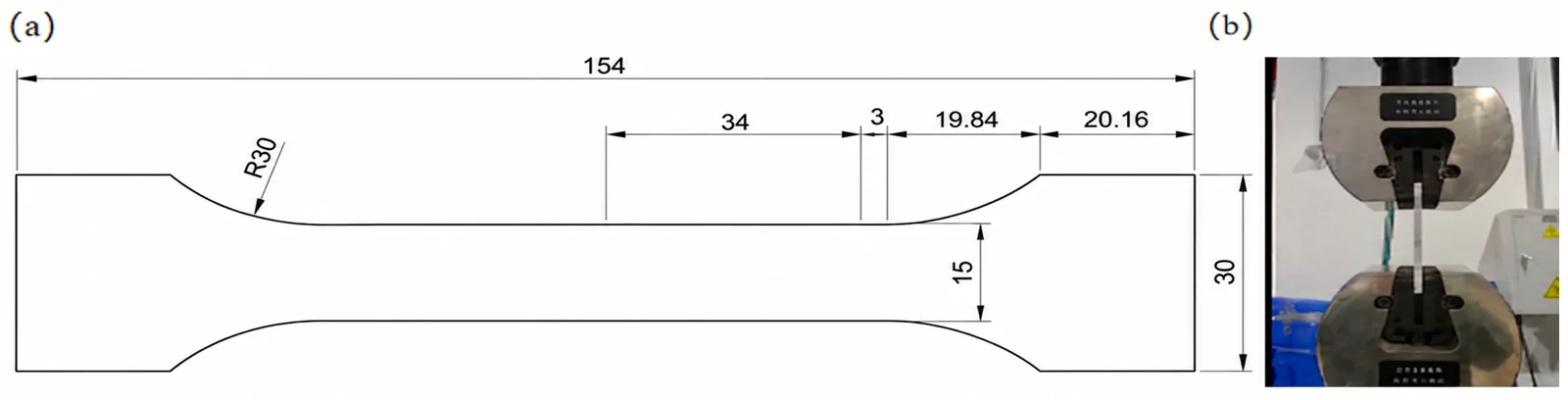
(**a**) Dimensions of the tensile specimen; (**b**) tensile test setup. All dimensions are in mm.

Tensile specimens were prepared by wire electrical discharge machining (WEDM) according to the geometry shown in Figure 2. Tensile tests were conducted using a DWD-50 electronic universal testing machine at a crosshead speed of 2 mm/min. Three independent specimens were tested for each specimen condition. Microhardness measurements were performed using an HVS-1000A automatic microhardness tester (Laizhou Huayin Testing Instrument Co., Ltd., Laizhou, China) under a load of 4.9 N and a dwell time of 10 s, with a measurement interval of 0.1 mm. For each specimen condition, three independent specimens were tested. Multiple indentations along the same measurement path were treated as spatial measurements within a specimen rather than independent experimental replicates.

**Figure 3:**
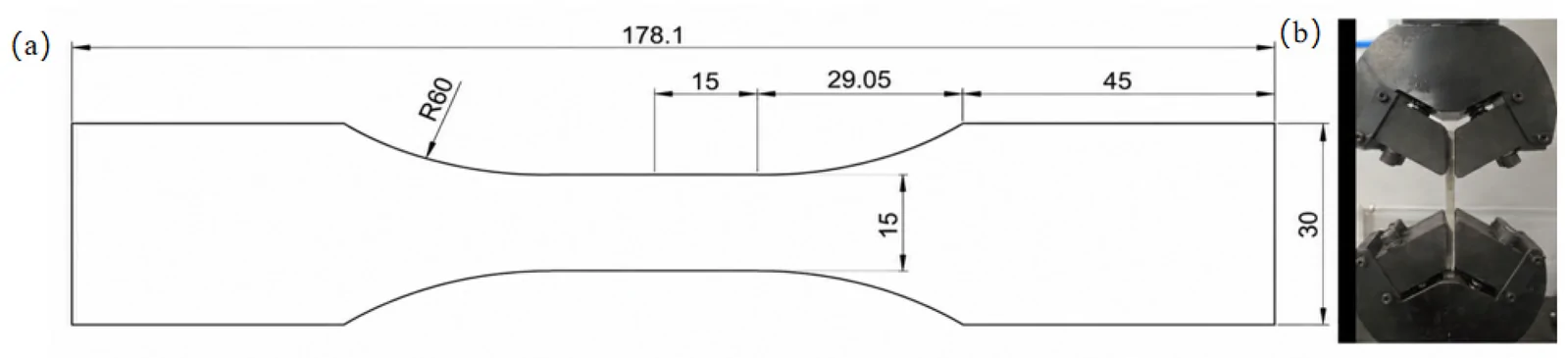
(**a**) Dimensions of the fatigue specimen; (**b**) fatigue test setup. All dimensions are in mm.

The geometry and dimensions of the fatigue specimens are shown in Figure 3. Fatigue tests were conducted using an HYG-50 high-frequency fatigue testing machine (Changchun Haoyuan, Changchun, China) under axial tension–tension stress-controlled loading. The stress ratio was R = 0.1, and the loading frequency ranged from 80 to 90 Hz. The S–N curve tests used six stress levels, with three independent specimens at each stress level (18 specimens for the inclined portion of each specimen condition). Additional specimens were tested using the staircase method for fatigue-strength evaluation. For statistical comparison of fatigue performance, fatigue lives obtained at the same stress amplitude were log_10_-transformed and analyzed using one-way analysis of variance (ANOVA), followed by Tukey’s post hoc test. A significance level of *p* < 0.05 was adopted. The three specimens tested at each stress amplitude constituted the independent replicates for the corresponding fatigue-life comparison.

## 3. Results and Discussion

The optimized laser-cut specimens, waterjet-cut specimens, and mechanically ground specimens were selected for comparative investigation. The effects of the three cutting processes on the cut-surface morphology, edge microstructure, microhardness, tensile properties, and high-cycle fatigue performance of 5083-H111 aluminum alloy were systematically analyzed. In combination with fatigue fracture characteristics, the influence of surface condition on crack initiation, propagation, and final failure was further discussed.

### 3.1. Cut-Surface Morphology Under Different Cutting Processes

**Figure 4:**
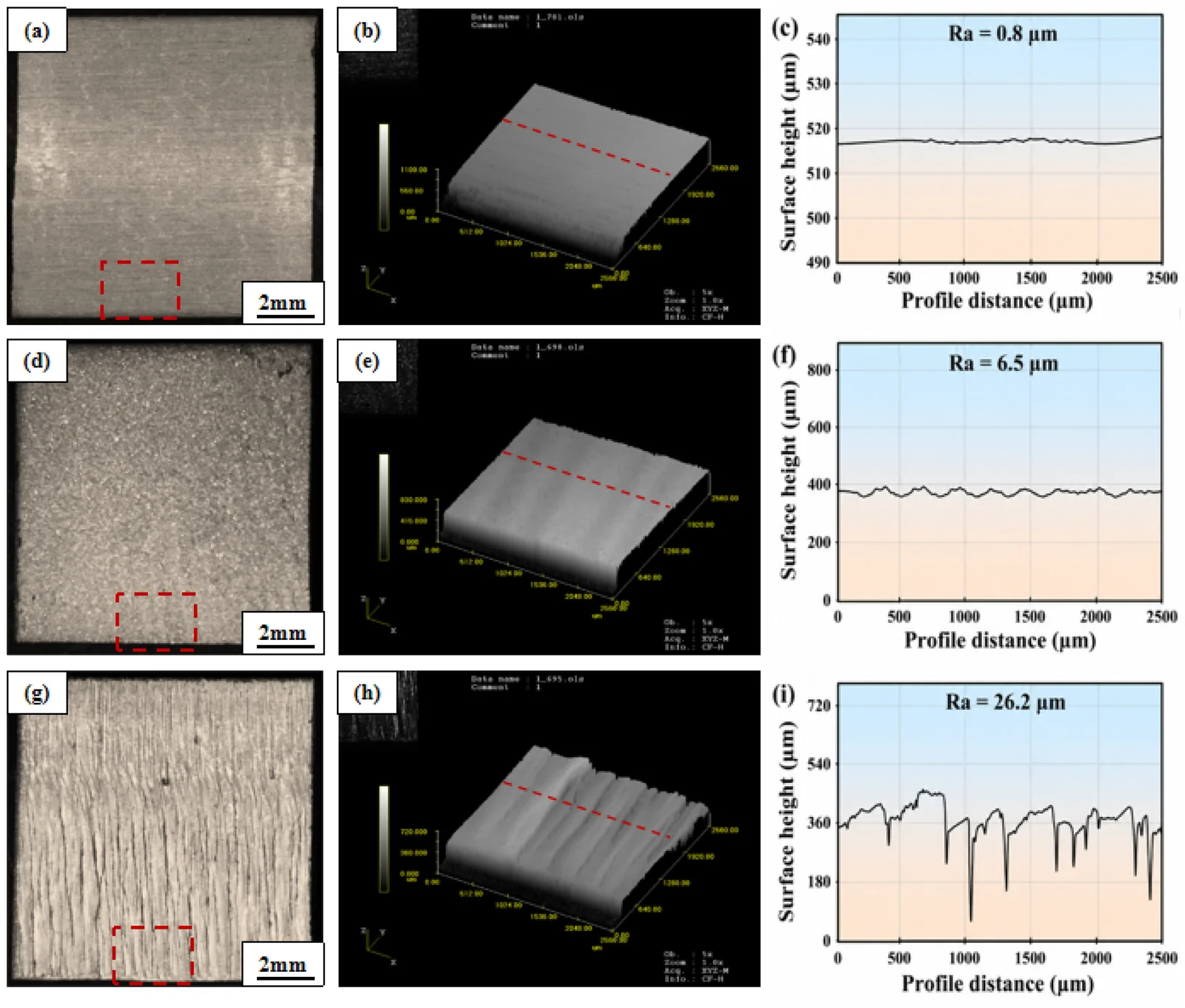
Macroscopic photographs, three-dimensional surface topographies, and two-dimensional surface profiles of different cut surfaces: (**a**–**c**) mechanical grinding; (**d**–**f**) abrasive waterjet cutting; (**g**–**i**) laser cutting.

Figure 4 presents the macroscopic morphology, three-dimensional topography, and surface profiles of the kerf surfaces produced by the three cutting processes. The mechanically ground specimen exhibited a smooth surface with few defects after grinding (Figure 4a). In waterjet cutting, material was removed by high-speed abrasive erosion and shearing without obvious melting. As a result, the cut surface was composed of dense microscopic impact pits and showed a relatively uniform matte appearance. Owing to the lagging and divergence of the waterjet during penetration through the plate, curved waviness formed near the lower part of the kerf (Figure 4d). During laser cutting, the material was locally melted and the molten metal was expelled by the assist gas. As shown by the typical microscopic morphology of the laser-cut surface in Figure 4g, the surface morphology was non-uniform along the plate thickness. The upper part of the kerf exhibited relatively smooth striations, whereas the lower part showed rough, inclined striations. This phenomenon can be attributed to the rapid cooling of the liquid aluminum alloy near the lower part of the kerf, which increased the melt viscosity. Under the action of the assist-gas flow, a backward viscous dragging effect was generated, ultimately resulting in the formation of rough inclined striations. Therefore, the region with the highest surface roughness in the lower part of the laser-cut surface was selected for comparison with the corresponding regions of specimens produced by the other cutting processes. The resulting Ra values were approximately 0.8 μm for mechanical grinding, 6.5 μm for waterjet cutting, and 26.2 μm for laser cutting. Accordingly, the value reported for the laser-cut specimens should be regarded as representative of the fatigue-relevant lower cut-edge region rather than as an average over the entire cut-surface thickness. This measurement strategy is also relevant to the fatigue behavior observed in this study. Fatigue cracks in the laser-cut specimens preferentially initiated from dross roots and valleys of surface striations near the lower cut edge, where local surface irregularities were most pronounced. These features act as geometric notches and intensify local stress concentration under cyclic loading. Thus, although the roughness varies along the plate thickness, the lower-region roughness is particularly important for evaluating fatigue-crack susceptibility.

### 3.2. Microstructure and Composition near the Cut Edge

#### 3.2.1. EBSD Analysis of the Cut-Edge Microstructure

**Figure 5:**
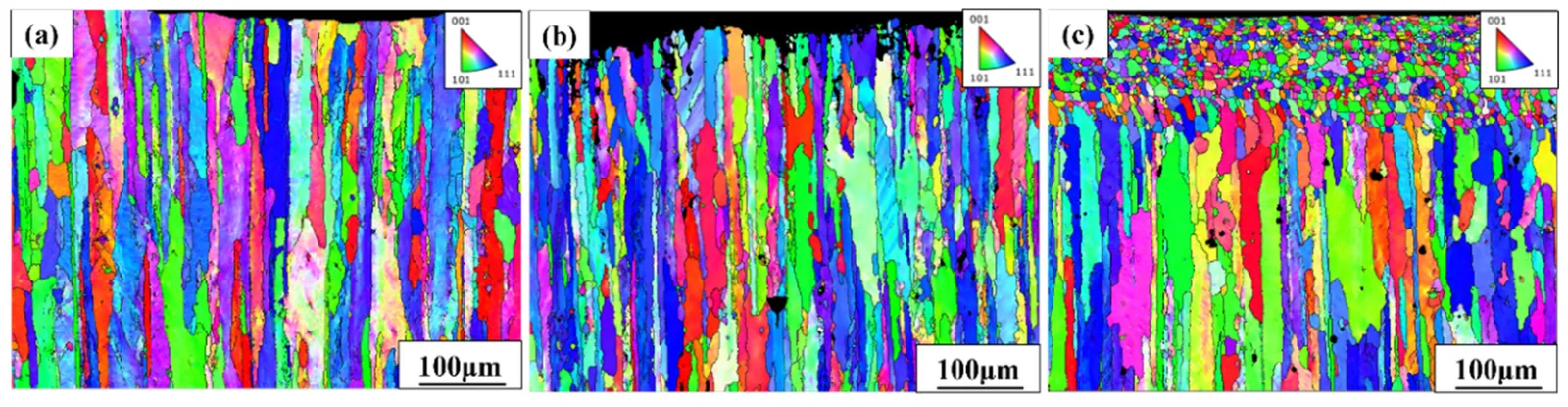
IPF maps of the kerf edges of specimens processed by different cutting methods: (**a**) machined specimen; (**b**) abrasive waterjet-cut specimen; (**c**) laser-cut specimen.

To investigate the effect of different cutting processes on the grain structure near the specimen edges, electron backscatter diffraction (EBSD) was used to characterize the microstructure near the kerf edges of specimens produced by the three cutting methods. The corresponding inverse pole figure (IPF) maps are shown in Figure 5. The mechanically ground specimen exhibited relatively intact grain morphology and retained the typical features of the rolled microstructure. In the waterjet-cut specimen, some locally unindexed dark regions were observed near the edge, which may be associated with severe plastic deformation and lattice distortion induced by abrasive impact, making crystallographic orientation indexing difficult. The laser-cut specimen exhibited a distinct layered structure near the edge. The laser-affected region mainly consisted of fine equiaxed grains formed by rapid solidification after melting, together with recrystallized regions induced by the laser thermal cycle. These regions collectively constituted the abnormal microstructural zone near the laser-cut surface, with a thickness of approximately 150 μm.

**Figure 6:**
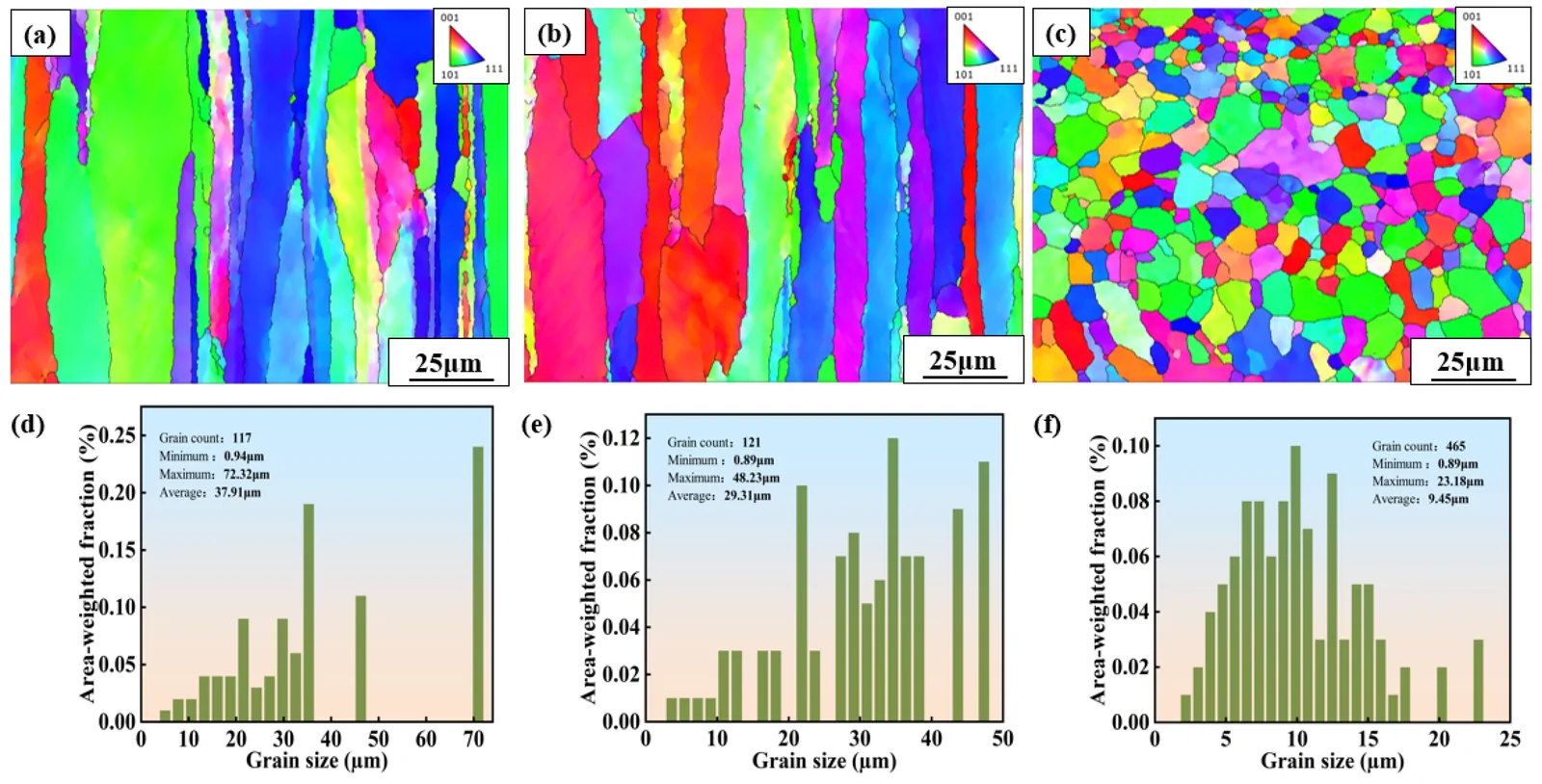
IPF maps and grain size distributions at the kerf edges of specimens processed by different cutting methods: (**a**,**d**) machined specimen; (**b**,**e**) abrasive waterjet-cut specimen; (**c**,**f**) laser-cut specimen.

To further compare the microstructural differences near the cut edges produced by different cutting processes, localized high-magnification observations were performed and the grain sizes were statistically analyzed, as shown in Figure 6. The average grain size near the edge of the mechanically ground specimen was 37.91 μm. For the waterjet-cut specimen, the average grain size decreased to 29.31 μm, representing a reduction of 22.7% compared with the mechanically ground specimen, which was mainly attributed to severe plastic deformation induced by high-velocity abrasive impact. The average grain size near the edge of the laser-cut specimen was only 9.45 μm, corresponding to a reduction of 75.1% relative to the mechanically ground specimen. Grain refinement induced by waterjet cutting is generally accompanied by work hardening, which contributes to an improvement in local strength. In contrast, the fine-grained microstructure near the laser-cut edge was mainly formed by high-temperature remelting followed by rapid solidification. Meanwhile, the original rolling-induced dislocations and deformation substructures were partially eliminated, accompanied by the precipitation of brittle phases and the formation of thermally induced microscopic defects, which could deteriorate the mechanical properties of this region.

**Figure 7:**
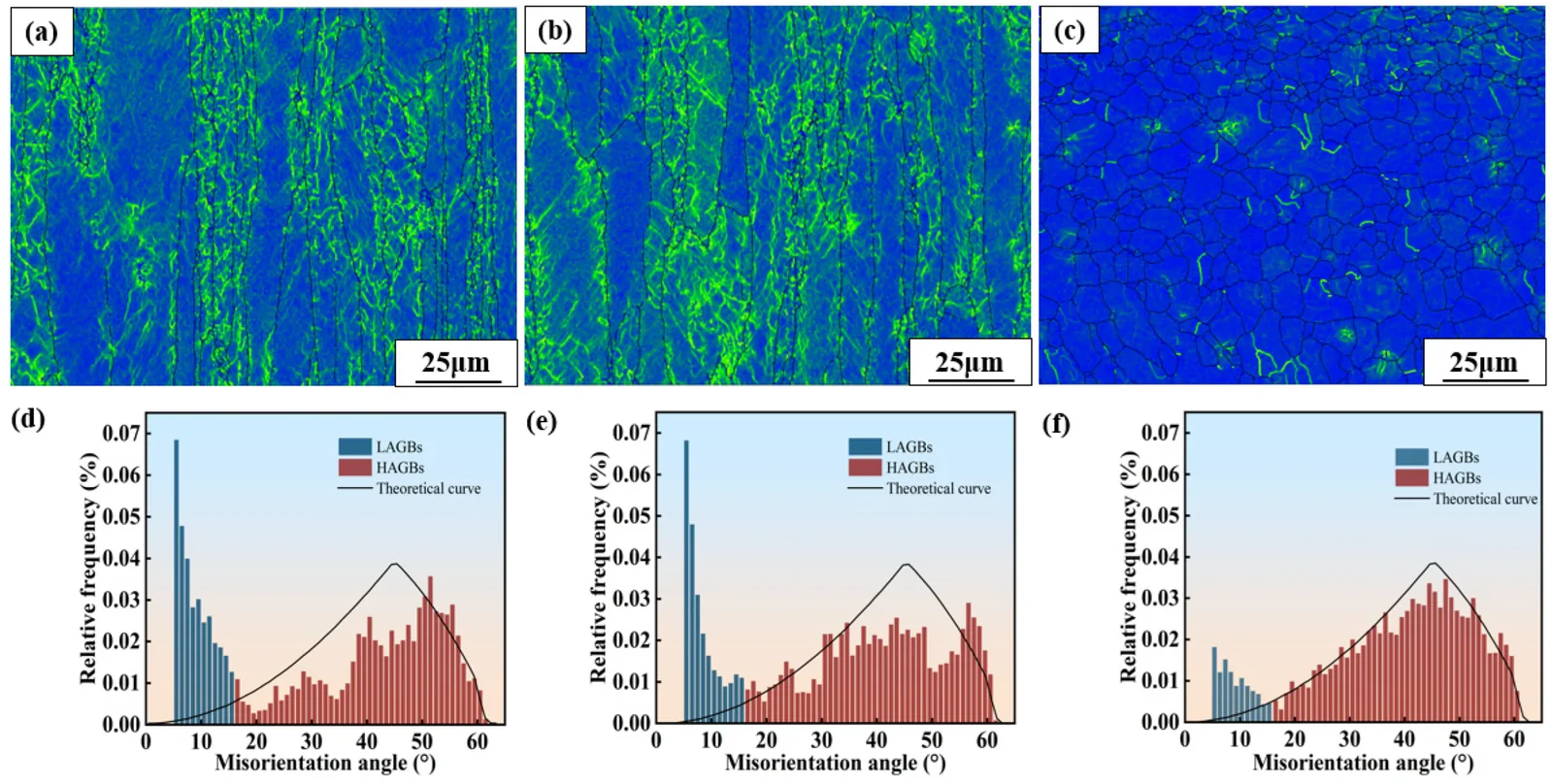
KAM maps and misorientation angle distributions at the kerf edges of specimens processed by different cutting methods: (**a**,**d**) machined specimen; (**b**,**e**) abrasive waterjet-cut specimen; (**c**,**f**) laser-cut specimen.

Figure 7a–c present the kernel average misorientation (KAM) maps near the cut edges of specimens produced by different cutting processes. The KAM value reflects local orientation gradients and lattice distortion, and can therefore be used as an indirect indicator of localized plastic deformation and stored strain. In the mechanically ground specimen, regions with relatively high KAM values were mainly distributed within grains and near grain boundaries, indicating that deformation characteristics associated with the original rolled microstructure were still retained after grinding. For the waterjet-cut specimen, the proportion of high-KAM regions increased further, suggesting that abrasive impact introduced additional plastic deformation and intensified lattice distortion in the near-surface region. In contrast, the laser-cut specimen exhibited generally lower KAM values, with high-KAM regions only locally distributed near grain boundaries. This indicates that the thermal cycle promoted recovery and localized recrystallization, thereby reducing the deformation features inherited from the original rolled microstructure.

The misorientation angle distributions are shown in Figure 7d–f. The fractions of low-angle grain boundaries (LAGBs) in the mechanically ground specimen and waterjet-cut specimens were 33.3% and 25.1%, respectively, indicating that a certain amount of deformation substructure was still retained in both specimens. In contrast, the fraction of LAGBs in the laser-cut specimen decreased to 10.9%, accompanied by a pronounced reduction in the low-angle misorientation peak and a corresponding increase in the fraction of high-angle grain boundaries (HAGBs). Combined with the KAM results, these observations indicate that the laser-induced thermal cycle transformed the near-edge microstructure from a work-hardened state toward recovery and recrystallization. This evolution reduced local lattice distortion while simultaneously weakening dislocation strengthening, which is consistent with the observed decreases in edge hardness and strength.

**Figure 8:**
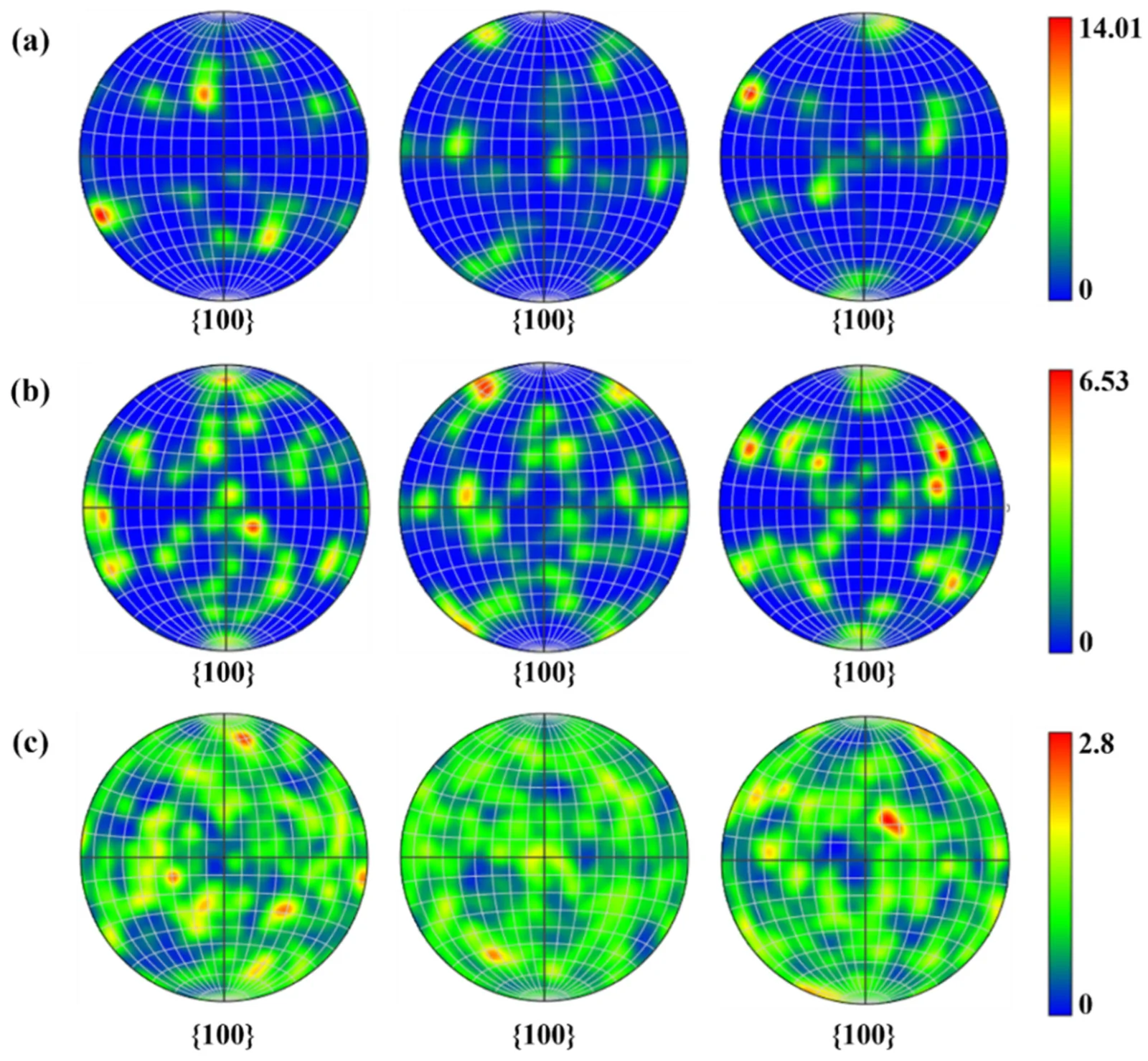
Pole figures at the kerf edges of specimens processed by different cutting methods: (**a**) machined specimen; (**b**) abrasive waterjet-cut specimen; (**c**) laser-cut specimen.

Figure 8 presents the pole figures near the cut edges of specimens produced by different cutting processes. The mechanically ground specimen retained a pronounced rolling texture, with concentrated pole distributions and a maximum texture intensity of 14.01, indicating a strong preferred crystallographic orientation. For the waterjet-cut specimen, the maximum texture intensity decreased to 6.53, accompanied by a more dispersed pole distribution, suggesting that the additional plastic deformation induced by abrasive impact partially weakened the original rolling texture. The laser-cut specimen exhibited a further decrease in the maximum texture intensity to 2.80, showing a distinct weak-texture characteristic and indicating that the thermal cycle altered the preferred orientation of the original rolled microstructure. Combined with the reductions in KAM values and the fraction of low-angle grain boundaries, these results indicate that the laser-induced thermal effect promoted recovery and recrystallization in the microstructure near the cut edge.

#### 3.2.2. XRD and Residual Stress Analysis of the Cut Surface

**Figure 9:**
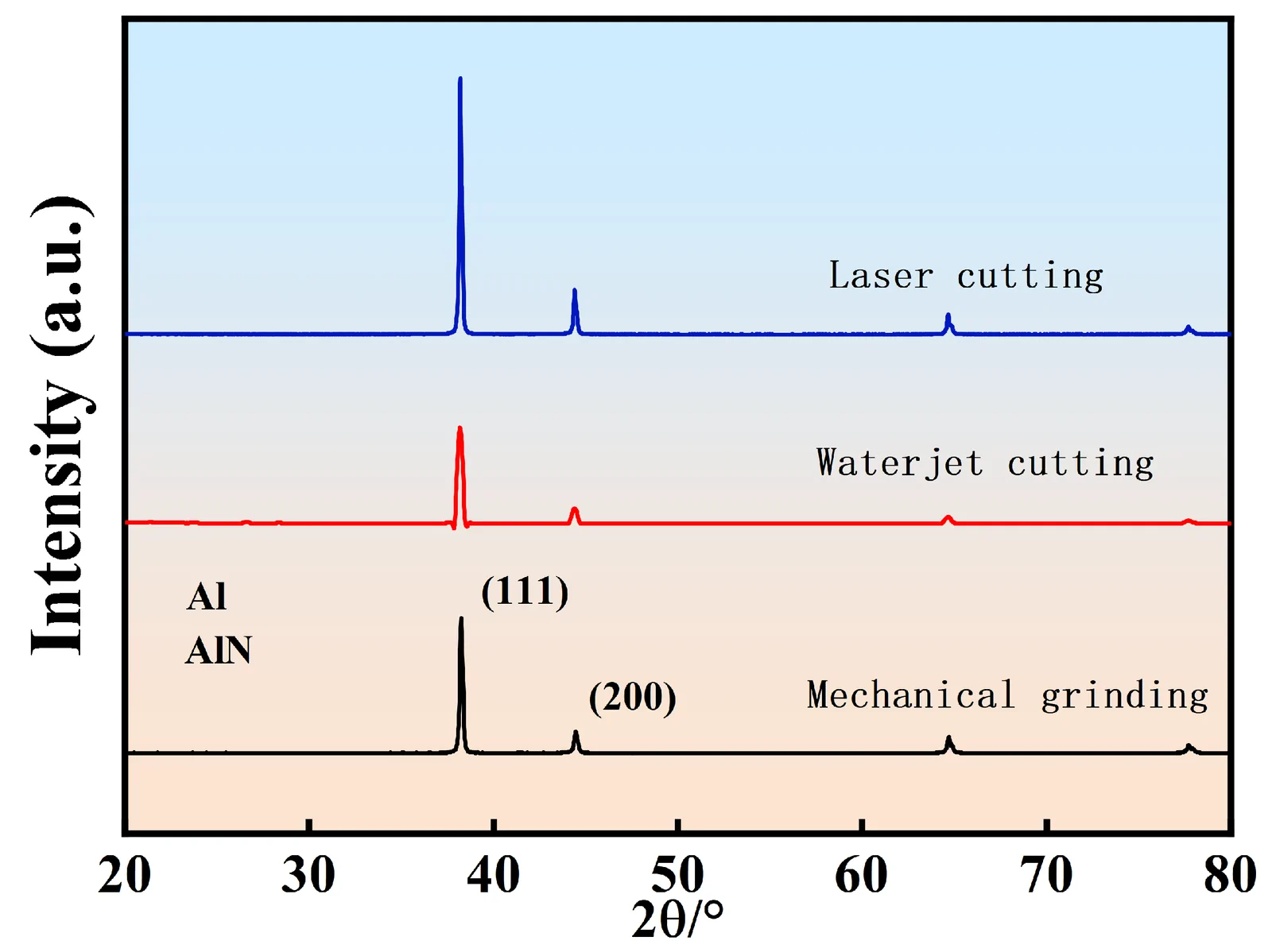
X-ray diffraction patterns of kerf surfaces produced by different cutting processes.

To investigate the effects of different cutting processes on the phase composition and crystallographic state of the kerf surface layer of 5083-H111 aluminum alloy, X-ray diffraction (XRD) analysis was performed on the cut surfaces, as shown in Figure 9. All three specimens exhibited the characteristic diffraction peak of the Al matrix (111) plane at approximately 2θ = 38.5°. Among them, the laser-cut specimen showed the highest peak intensity, followed by the mechanically ground specimen, whereas the waterjet-cut specimen exhibited the lowest intensity. These differences may be related to variations in near-surface lattice distortion and microstructural state induced by the different cutting processes. The high-temperature thermal cycle during laser cutting promoted recovery and recrystallization near the kerf edge, resulting in fine equiaxed grains with relatively high crystallographic integrity and, consequently, an enhanced diffraction peak intensity. In contrast, high-velocity abrasive impact during waterjet cutting introduced a high density of dislocations and severe lattice distortion into the surface layer, leading to diffraction peak broadening and reduced peak intensity. In addition, no obvious AlN phase was detected on the surface of the aluminum alloy after laser cutting in a nitrogen atmosphere.

**Figure 10:**
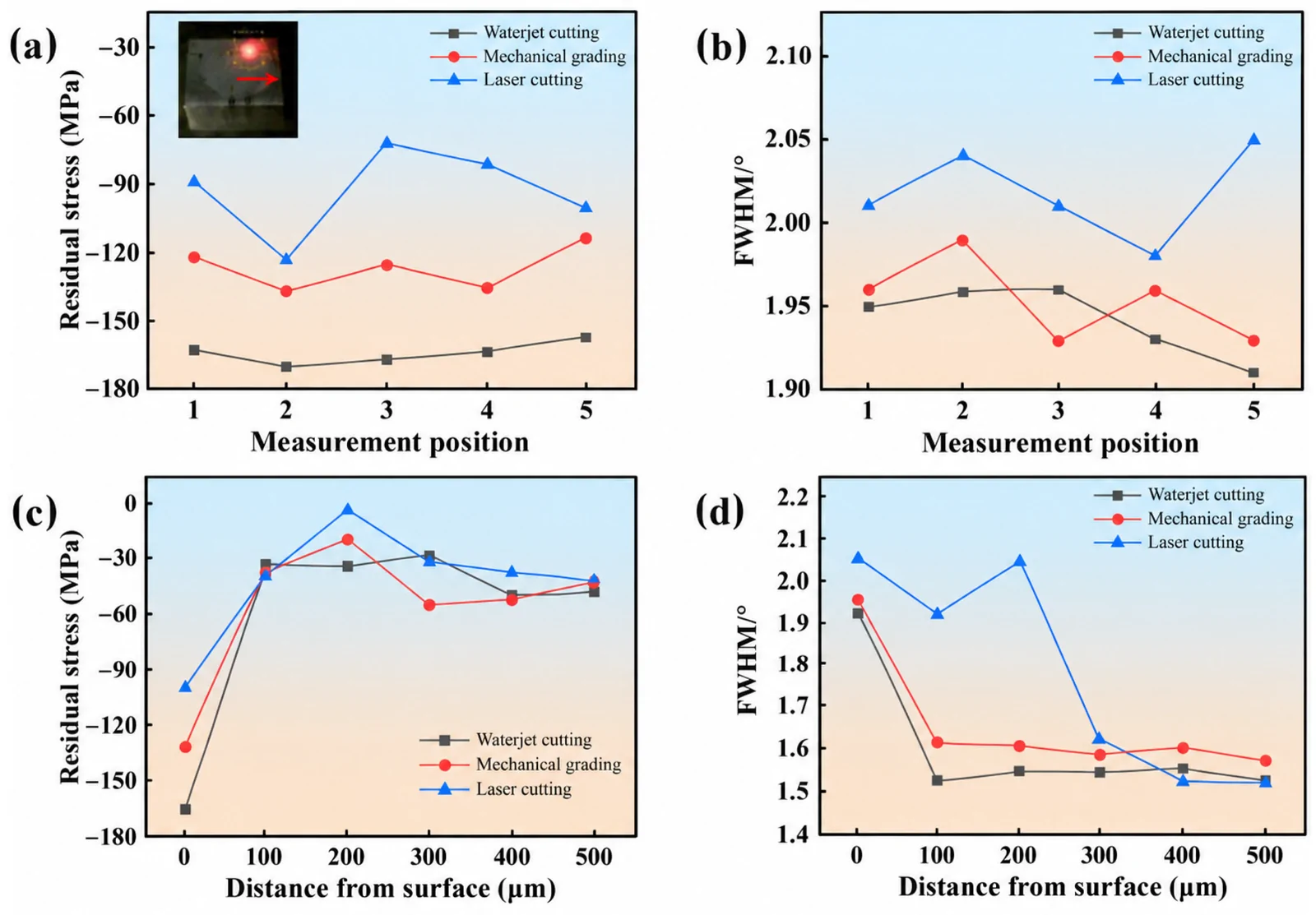
Residual stresses and full widths at half maximum (FWHM) of diffraction peaks for different kerf surfaces: (**a**) surface residual stress at five measurement positions; (**b**) corresponding FWHM values; (**c**) residual stress as a function of depth from the cut surface; (**d**) corresponding FWHM values. Positions 1–5 in (**a**) and (**b**) represent five measurement locations along the horizontal direction of the cut surface.

Residual stress is an important factor affecting component deformation, cracking, and fatigue life, while residual tensile stress generally promotes fatigue crack initiation and propagation. To investigate the effect of different cutting processes on the near-surface stress state of 5083-H111 aluminum alloy, residual stresses were measured by X-ray diffraction at five positions along the horizontal direction of the cut surface, and the corresponding full width at half maximum (FWHM) values of the diffraction peaks were obtained simultaneously, as shown in Figure 10a,b. Residual stress reflects the distribution of elastic strain within the material after processing, whereas FWHM can be used to characterize structural features such as lattice distortion, grain refinement, and defect density. The mechanically ground specimen exhibited a surface residual compressive stress of approximately −130 MPa and an FWHM of about 1.93°. High-velocity abrasive impact during waterjet cutting introduced severe plastic deformation into the surface layer, increasing the residual compressive stress to approximately −170 MPa and slightly increasing the FWHM to 1.96°, indicating increased lattice distortion and defect density near the surface. In contrast, the laser-cut specimen was subjected to high-temperature remelting and thermal cycling, which promoted recovery and recrystallization of the original rolled microstructure and relaxed the surface residual compressive stress to approximately −90 MPa. Owing to the relatively high surface roughness, some fluctuation was observed in the measured residual stress. The FWHM increased to approximately 2.02°, which may be associated with the fine equiaxed grains and local lattice distortion within the thermally affected region.

The depth-dependent measurements showed that all three specimens exhibited relatively high residual compressive stresses near the surface, which rapidly decreased to approximately −30 MPa at a depth of about 50 μm, indicating that the compressive-stress-affected layer was relatively shallow. For the waterjet-cut specimen, the FWHM remained high only in the near-surface region and then rapidly decreased to approximately 1.5°, suggesting that the influence of plastic deformation was limited to a shallow depth. The FWHM of the laser-cut specimen remained approximately 2.0° at a depth of about 150 μm below the surface, indicating that the laser-induced abnormal microstructural zone extended to a depth of approximately 150 μm, consistent with the microstructural observations.

### 3.3. Mechanical Properties of Laser-Cut Specimens

**Figure 11:**
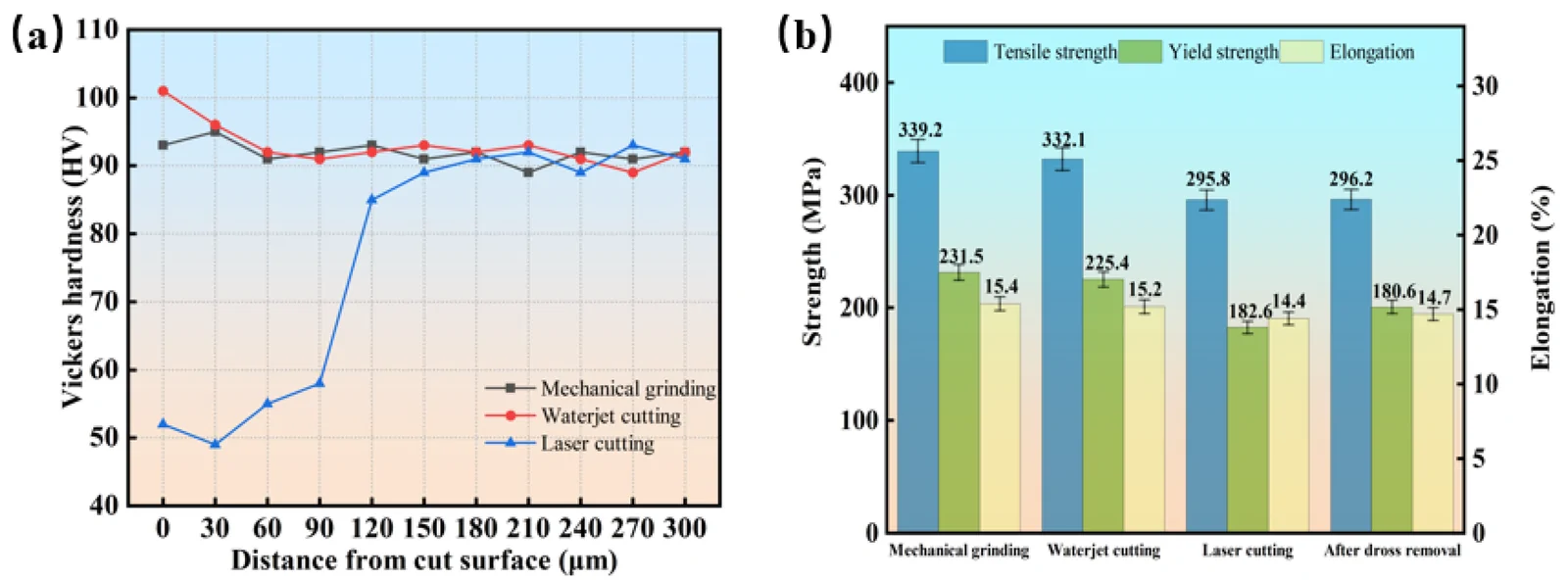
Properties of specimens processed by different cutting methods: (**a**) microhardness distributions; (**b**) tensile properties.

Figure 11a shows the microhardness distributions near the cut edges of specimens produced by the three cutting processes. The edge hardness of the mechanically ground specimen was approximately 90 HV. For the waterjet-cut specimen, high-velocity abrasive impact induced near-surface plastic deformation, increased dislocation density, and work hardening, resulting in an increase in edge hardness to approximately 100 HV. In contrast, pronounced softening was observed in the laser-cut specimen, with the hardness decreasing to approximately 50–60 HV. The extent of the softened zone was generally consistent with that of the abnormal microstructural region. The laser-induced thermal cycle caused localized remelting near the cut edge and promoted recovery and recrystallization in the adjacent region, thereby weakening the dislocation-strengthening effect inherited from the original rolled microstructure. Consequently, the cut edge became more susceptible to localized plastic deformation and crack initiation, forming a mechanically weakened region.

As shown in Figure 11b, the mechanically ground specimen exhibited the highest ultimate tensile strength and yield strength, reaching 339.2 MPa and 231.5 MPa, respectively. The waterjet-cut specimen showed slightly lower values of 332.1 MPa and 225.4 MPa, respectively. For the laser-cut specimen without dross removal, the ultimate tensile strength and yield strength decreased to 295.8 MPa and 182.6 MPa, respectively. After dross removal, these values changed to 296.2 MPa and 180.6 MPa, respectively. The waterjet-cut surface exhibited a roughness of Ra = 6.5 μm, and the associated microscopic surface undulations induced local stress concentration, resulting in slightly lower strength than that of the mechanically ground specimen. In contrast, the laser-cut specimens were simultaneously affected by the notch effects associated with surface striations and dross, edge microstructural softening, and defects such as micropores, which reduced the local load-bearing capacity. Changes in precipitates and grain-boundary characteristics may also have contributed to the reduction in mechanical properties. Although dross removal alleviated stress concentration at the lower edge, the surface striations and thermally induced microstructural degradation remained, resulting in only limited improvement in tensile performance.

**Figure 12:**
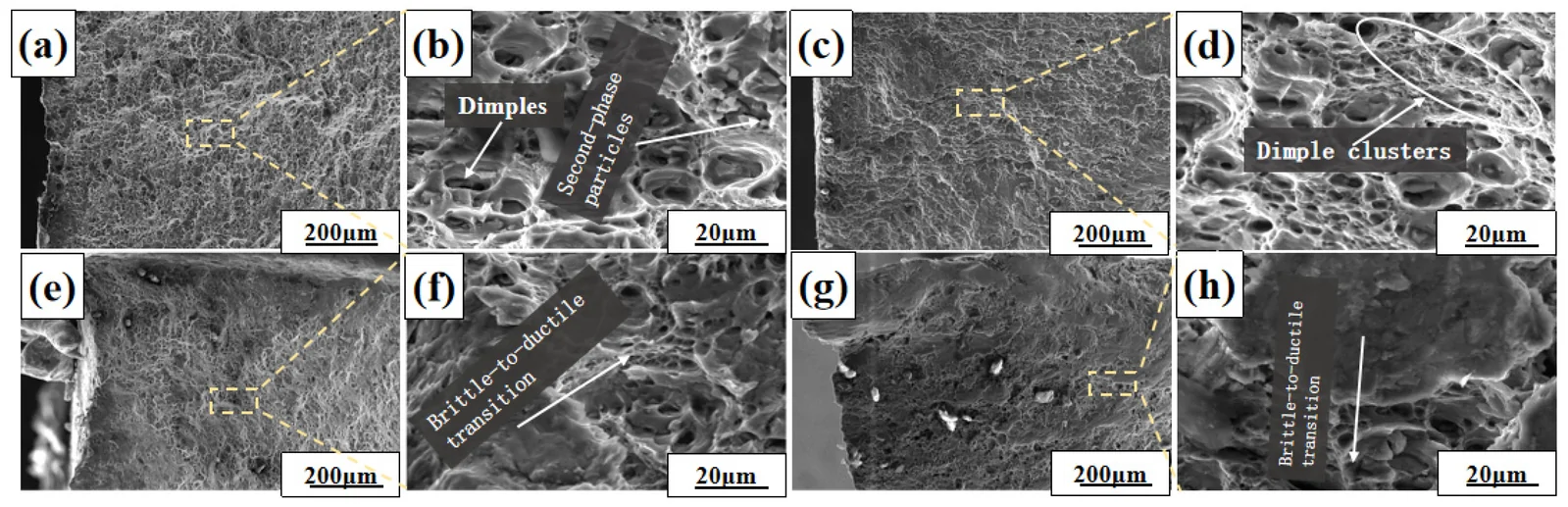
Microscopic morphologies of tensile fracture surfaces: (**a**,**b**) machined specimen; (**c**,**d**) abrasive waterjet-cut specimen; (**e**,**f**) laser-cut specimen without dross removal; (**g**,**h**) laser-cut specimen after dross removal.

Figure 12 presents the SEM morphologies of the tensile fracture surfaces of specimens produced by different cutting processes. As shown in Figure 12a–d, the mechanically ground specimen and waterjet-cut specimens exhibited typical ductile fracture characteristics, with numerous spherical dimples distributed over the fracture surfaces. Second-phase particles were observed within some of the dimples. During tensile deformation, these particles can act as nucleation sites for microvoids. Interfacial debonding between the particles and the aluminum matrix leads to the formation of initial microvoids, which subsequently grow and coalesce, eventually forming the macroscopic fracture surface. Regardless of whether the dross was removed, relatively flat and bright regions were observed near the fracture edges of the laser-cut specimens, indicating local brittle fracture characteristics, as shown in Figure 12e,g. On the one hand, rapid melting and solidification near the kerf edge caused elemental segregation, resulting in the precipitation of a small amount of Si-containing impurity phases. On the other hand, microcracks formed during solidification could act as preferential fracture initiation sites and propagate rapidly by cleavage or intergranular fracture under tensile loading. When the cracks propagated into the more ductile base-material region, the fracture mechanism gradually transitioned to microvoid-coalescence-dominated ductile fracture, producing numerous dimples, as shown in Figure 12f,h. Therefore, the deterioration in the mechanical properties of the laser-cut specimens was closely associated with the abnormal near-edge microstructure, brittle phases, and solidification defects.

### 3.4. High-Cycle Fatigue Performance

**Figure 13:**
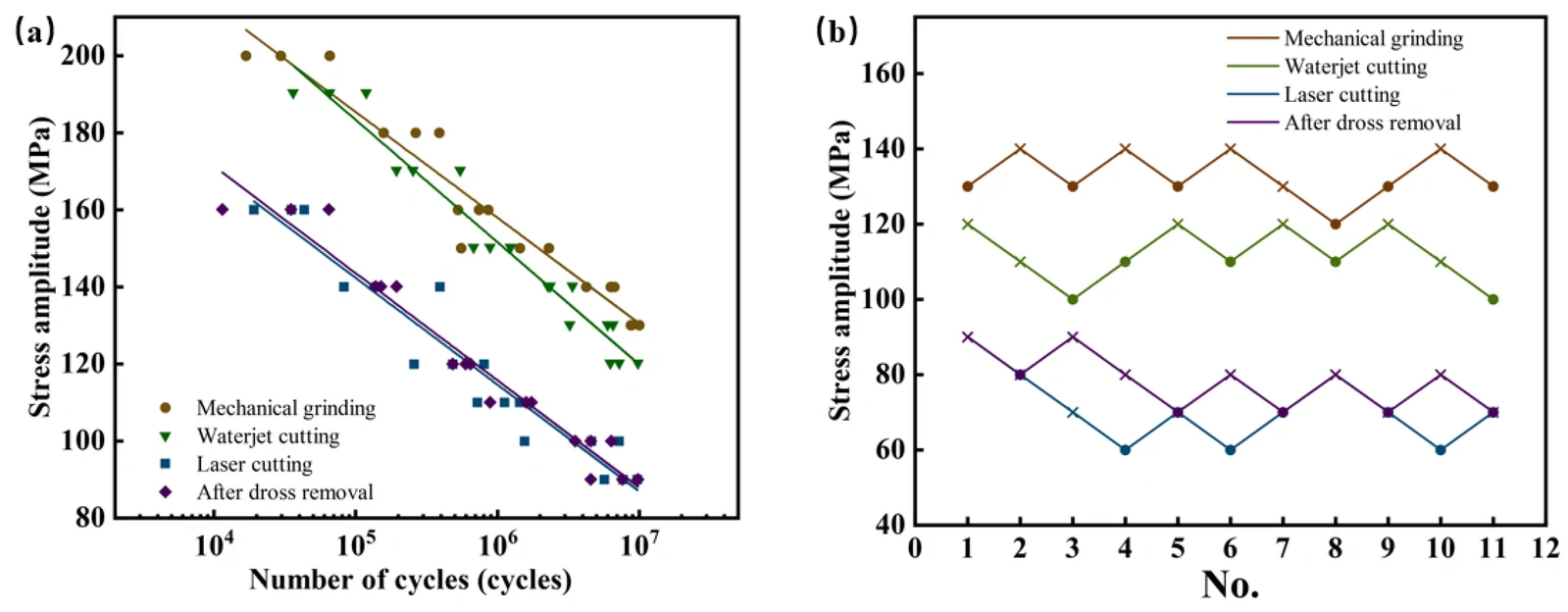
Fatigue life (S–N) curves of specimens processed by different methods: (**a**) S–N curves; (**b**) staircase-test data.

Aluminum alloys and their products generally do not exhibit a well-defined fatigue limit; therefore, the fatigue strength corresponding to a specified number of loading cycles is commonly used to evaluate their fatigue performance. Figure 13a shows the S–N curves of the mechanically ground, waterjet-cut, laser-cut without dross removal, and laser-cut after dross removal specimens. The mechanically ground specimens exhibited the best fatigue performance, followed by the waterjet-cut specimens, whereas the laser-cut specimens showed markedly shorter fatigue lives. At the same stress level, dross removal prolonged the fatigue life of the laser-cut specimens, although the improvement was limited. To further evaluate the statistical significance of the differences in fatigue performance, fatigue lives obtained at a common stress amplitude of 160 MPa were compared among the mechanically ground, waterjet-cut, and laser-cut specimens without dross removal. Because fatigue life is typically analyzed on a logarithmic scale, the cycle numbers were transformed to log10(N) before statistical analysis. One-way analysis of variance (ANOVA) revealed a significant effect of the cutting process on fatigue life (F(2,6) = 33.01, *p* < 0.001). Tukey’s post hoc test showed that the fatigue life of the laser-cut specimens was significantly lower than those of the waterjet-cut specimens (*p* = 0.0026) and mechanically ground specimens (*p* = 0.0006). Although the mechanically ground specimens exhibited a higher mean fatigue life than the waterjet-cut specimens, the difference between these two groups was not statistically significant at this stress level (*p* = 0.216). These statistical results further confirm the pronounced deterioration in fatigue resistance caused by laser cutting.

The mean fatigue strength was determined using the modified staircase method described in Section 7.4 of the Chinese national standard GB/T 24176-2009 [25]. The standard deviation of fatigue strength was first calculated from the experimentally obtained S–N curve data, and the stress increment used in the staircase method was selected to be close to this standard deviation. The number of specimens required for the horizontal portion of the fatigue curve should satisfy the following relationship:

(1) $$\frac{n_{2}}{n_{1}}=\frac{l+1}{2l-1}$$

where *n*_1_ is the number of specimens in the inclined portion, *n*_2_ is the number of specimens in the horizontal portion, and *l* is the number of stress levels in the inclined portion.

With 18 specimens in the inclined portion and six stress levels, the required number of specimens for the staircase method was calculated to be 11 according to Equation (1). Figure 13b presents the staircase-test data for the four specimen conditions.

The mean fatigue strength was calculated using the following equation:

(2) $${\hat{\mu }}_{y}=\frac{\sum_{i=2}^{n+1}S_{i}}{n}$$

The lower limit of the fatigue strength for the overall sample population was estimated using the following equation:

(3) $${\hat{y}}_{\left(p,1-\alpha \right)}={\hat{\mu }}_{y}-k_{\left(p,1-\alpha ,\nu \right)}{\hat{\sigma }}_{y}$$

where *ν* is the number of degrees of freedom associated with the standard deviation estimated from the S–N test data (*ν* = *n* − 2).

Comparison of the lower-bound fatigue strengths of the four specimen conditions at 1 × 10^7^ cycles shows that the mechanically ground specimens exhibited the highest fatigue strength, reaching 119.18 MPa, followed by the waterjet-cut specimens at 99.21 MPa. The laser-cut specimens without dross removal showed the lowest fatigue strength, at 60.19 MPa, whereas the value increased to 67.64 MPa after dross removal. Overall, the fatigue strength of the laser-cut specimens was markedly lower than that of the waterjet-cut and mechanically ground specimens.

After surface grinding, the mechanically ground specimens exhibited relatively smooth cut edges with fewer defects, resulting in the best fatigue performance. Waterjet cutting is essentially a cold-working process and produces negligible heat-affected zones. Although some surface waviness was present, the relatively low surface roughness and good overall surface integrity provided greater resistance to fatigue crack initiation. Consequently, the fatigue strength of the waterjet-cut specimens reached 83.24% of that of the mechanically ground specimens. In contrast, the laser-cut specimens contained periodic surface striations, bottom-edge dross, softened edge microstructures, and thermal cracks. Under cyclic loading, these features readily induced local stress concentration and promoted fatigue crack initiation, leading to a pronounced reduction in fatigue strength. After dross removal, the fatigue strength of the laser-cut specimens increased by 12.38%, indicating that removal of dross can partially alleviate the notch effect and improve fatigue performance. However, because the periodic surface striations and microstructural degradation within the heat-affected zone remained, the overall improvement was limited.

### 3.5. Fatigue Fracture and Crack Initiation Mechanisms

#### 3.5.1. Macroscopic Analysis of Fatigue Fracture

**Figure 14:**
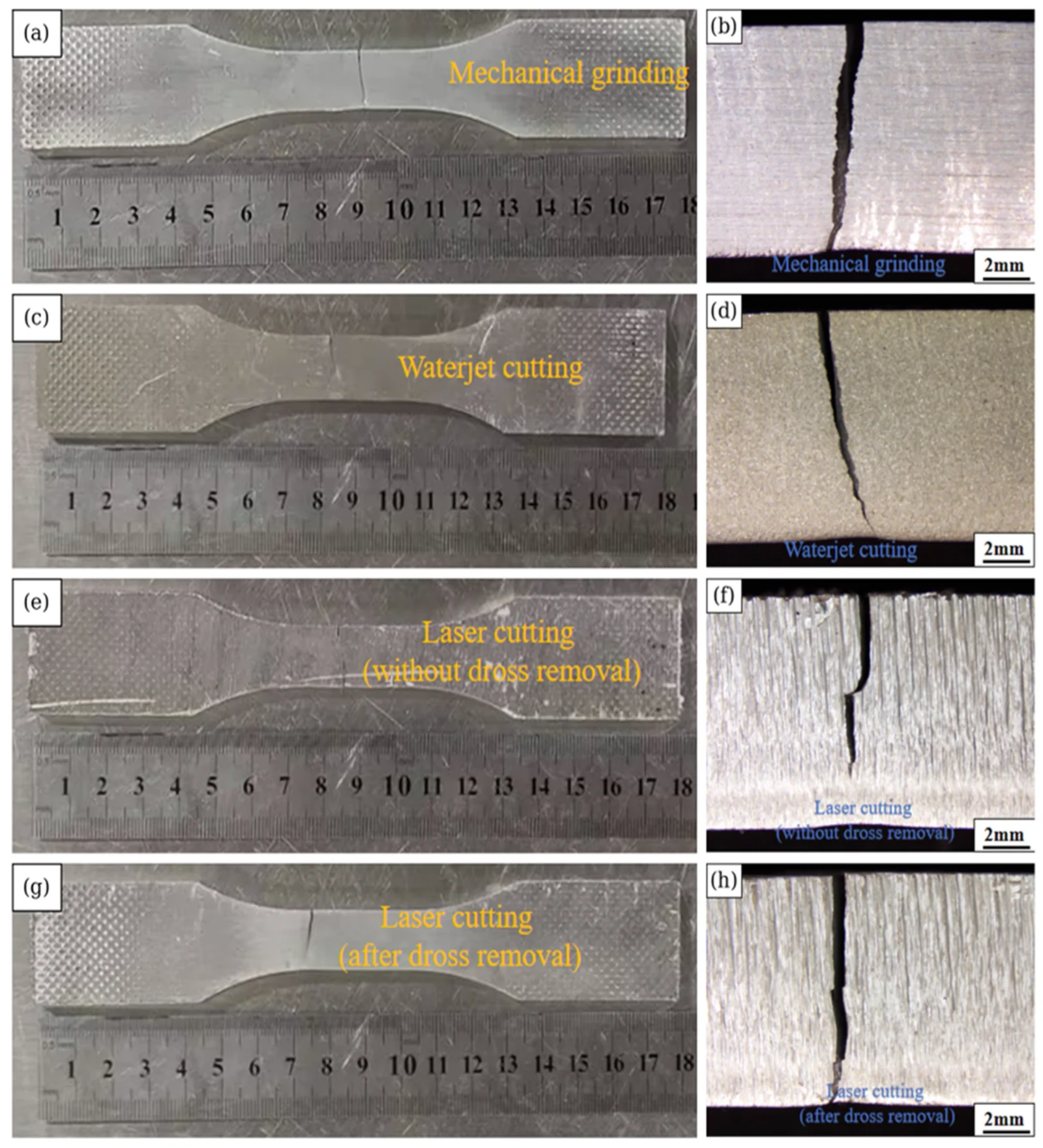
Macroscopic crack propagation paths of specimens prepared using different cutting processes: (**a**,**b**) mechanical grinding; (**c**,**d**) waterjet cutting; (**e**,**f**) laser cutting without dross removal; and (**g**,**h**) laser cutting after dross removal. The left and right columns show the crack paths on the specimen surface and along the thickness direction, respectively.

Figure 14 shows the macroscopic crack propagation paths along the specimen surface and through the thickness for the four specimen conditions under the same cyclic stress level. Both the mechanically ground specimen and waterjet-cut specimens were dominated by a single main crack, which propagated approximately perpendicular to the loading direction, as shown in Figure 14a,c. In contrast, two independent cracking regions were observed in the laser-cut specimen without dross removal, as shown in Figure 14e, indicating that multiple surface defects may have participated in fatigue crack initiation. The notch effects associated with surface striations and dross roots can generate local stress concentrations, promoting independent crack growth from different locations. After dross removal, the specimen was mainly characterized by a single dominant crack, as shown in Figure 14g, suggesting that reducing the influence of dross and the associated root micro-notches can decrease the tendency for multiple crack initiation.

Through the thickness, the crack path in the mechanically ground specimen was relatively straight, whereas only slight local curvature was observed in the waterjet-cut specimen, as shown in Figure 14b,d. The laser-cut specimen without dross removal exhibited pronounced crack deflection, while the degree of deflection decreased after dross removal, although local deviations remained, as shown in Figure 14f,h. This behavior may be related to the geometric notches formed by the surface striations and to microstructural heterogeneity near the cut edge. Stress concentration at the striation valleys, together with changes in their local orientation, can alter the local stress state at the crack tip and promote crack deflection. Therefore, dross removal can improve the cracking behavior to some extent; however, residual surface striations and edge microstructural alterations continue to affect crack propagation, consistent with the limited improvement observed in fatigue performance.

The driving force for fatigue crack propagation is governed by the stress intensity factor, KK, at the crack tip. In the laser-cut specimens, surface cracks predominantly propagated along planes approximately perpendicular to the tensile stress direction, and the fatigue failure mode may shift from being controlled by a single dominant crack to competitive propagation among multiple cracks. During crack propagation through the thickness, asymmetric geometric notches formed by the surface striations can introduce Mode II (sliding mode) and/or Mode III (tearing mode) stress intensity factors at the crack tip, resulting in mixed-mode loading and causing the crack to deviate from its original propagation plane. Surface integrity is therefore a critical factor governing fatigue failure behavior. Improving the machined surface quality and eliminating critical initial defects are beneficial for enhancing the fatigue performance of structural components.

#### 3.5.2. Fatigue Fracture Morphology of Laser-Cut Specimens

**Figure 15:**
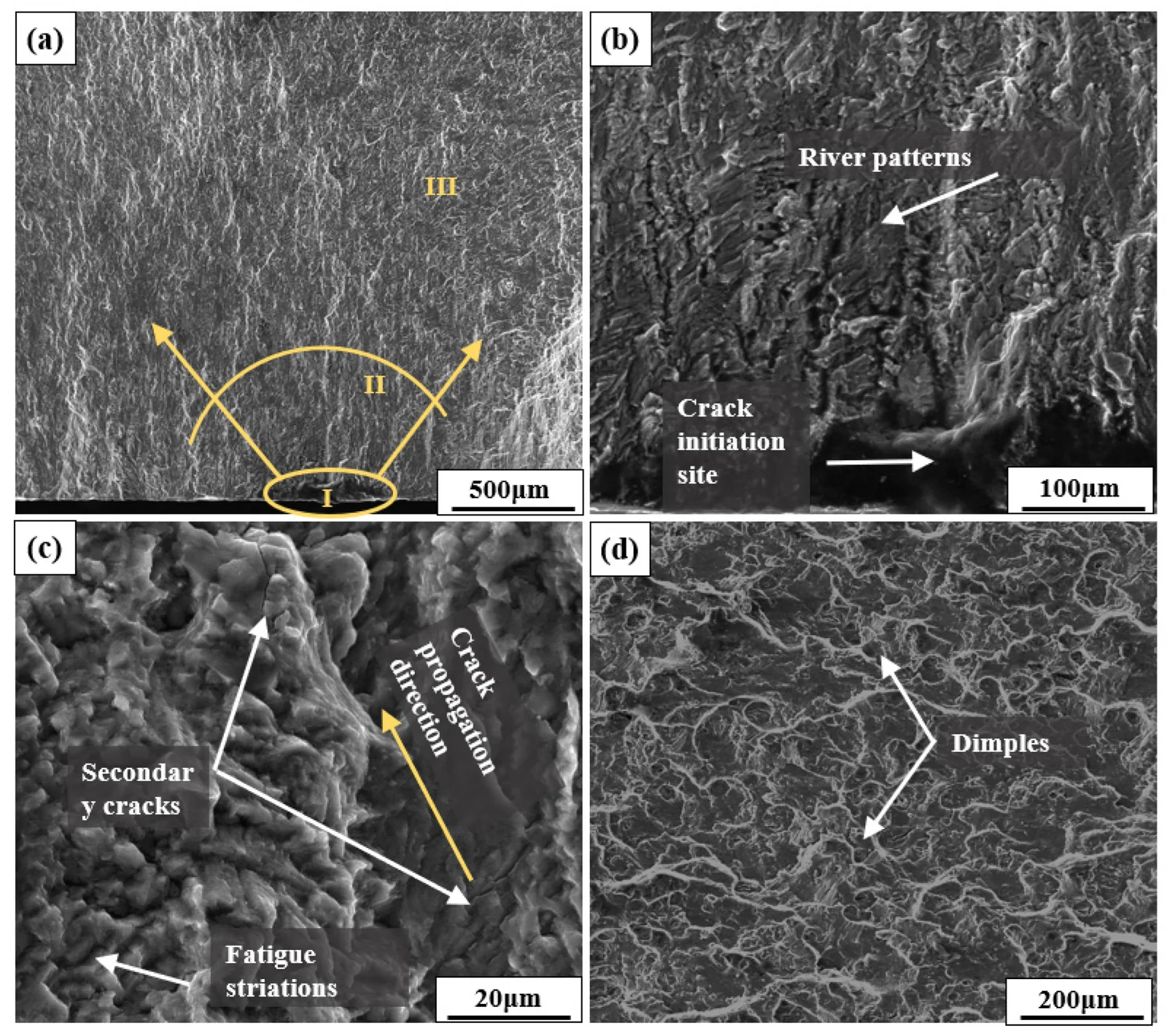
Fatigue fracture morphologies of the laser-cut specimen: (**a**) overall fracture morphology; (**b**) crack initiation region; (**c**) crack propagation region; (**d**) final fracture region.

Figure 15a presents a typical fatigue fracture surface of the laser-cut specimen after dross removal. The fracture surface can be divided into three characteristic regions, as marked in Figure 15a: Region I is the fatigue crack initiation region, Region II is the stable crack propagation region, and Region III is the final fracture region. As shown in Figure 15b, fatigue cracks mainly initiated from thermal cracks located within the heat-affected zone of the laser-cut edge. Under cyclic loading, these defects induced local stress concentration and acted as fatigue crack initiation sites, from which the cracks propagated outward in a fan-shaped pattern. Pronounced river patterns were also observed in the crack initiation region, and their convergence direction pointed toward the crack origin. Figure 15c shows typical fatigue striations in the stable crack propagation region, accompanied by secondary cracks oriented approximately perpendicular to the main crack propagation direction. In the final fracture region shown in Figure 15d, numerous dimples of non-uniform size were observed; however, their density and depth were lower than those observed on the tensile fracture surfaces. A small number of cleavage steps were also locally present, indicating that the final fracture of the laser-cut specimen exhibited a mixed brittle–ductile fracture mode.

**Figure 16:**
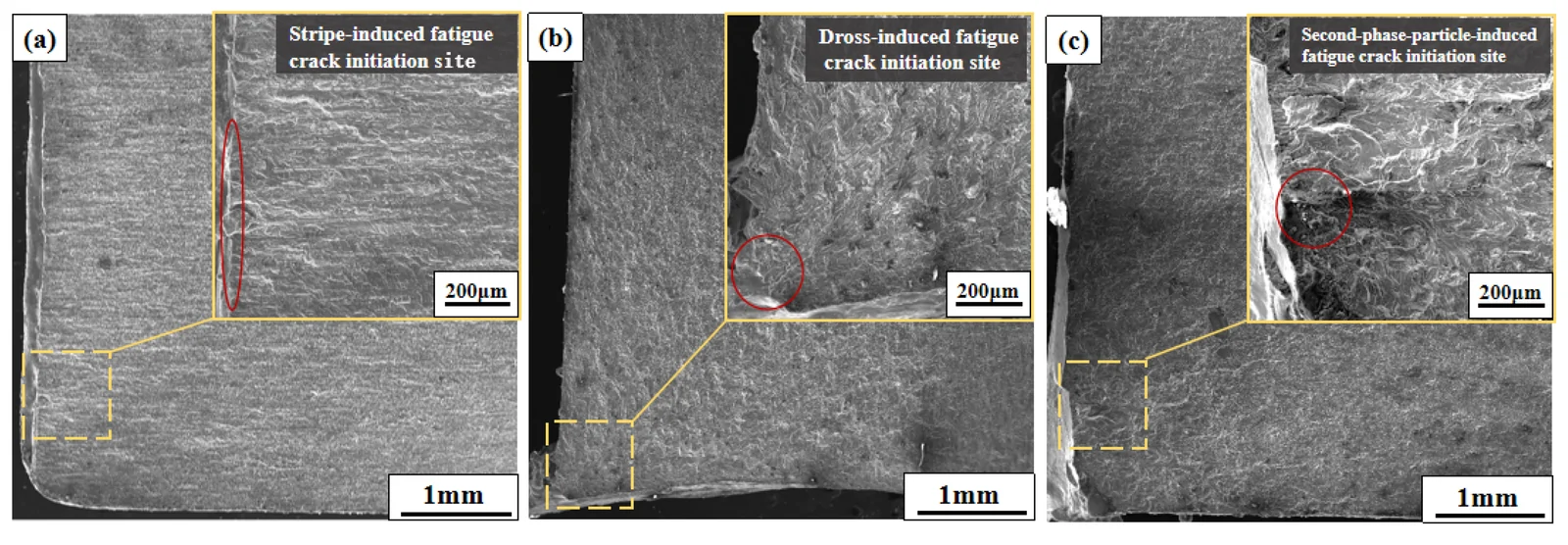
Types of fatigue crack initiation sites in laser-cut specimens: (**a**) stripe-induced; (**b**) dross-induced; (**c**) second-phase-particle-induced.

Comparison of the fatigue fracture surfaces of multiple laser-cut specimens revealed that fatigue cracks consistently initiated from the specimen surface, although the specific initiation sites varied with the surface condition, as shown in Figure 16. For specimens without dross removal, fatigue cracks predominantly initiated at the dross root along the lower cut edge. After dross removal, crack initiation occurred mainly at the periodic surface striations and, in some specimens, at microscopic surface defects.

Figure 16a presents a typical morphology of fatigue crack initiation and propagation along the surface striations generated by laser cutting. The cracks propagated both laterally along the striation direction and inward from the striation roots into the specimen, indicating that the valleys of the periodic striations readily act as local stress concentrators and promote fatigue crack initiation. Figure 16b shows a typical fracture morphology in which dross acted as the crack initiation source. The dross generally exhibited sharp burr-like or irregular droplet-like morphologies, and the abrupt geometric transition at its root formed a natural notch that generated pronounced stress concentration under cyclic loading. In addition, because the dross was formed by resolidification of molten metal, its internal microstructure was relatively loose and heterogeneous and may contain defects such as microshrinkage cavities, pores, and solidification cracks. These features make the dross root a preferential site for fatigue crack initiation. Figure 16c illustrates a failure mode in which fatigue cracks initiated from microscopic surface defects. Thermal cracks and second-phase particles within the heat-affected zone can serve as preferential sites for crack nucleation under cyclic loading. With continued crack propagation, these defects ultimately contributed to fatigue fracture.

## 4. Fatigue Failure Mechanism

Based on the preceding results, the differences in fatigue performance among the three cutting conditions were mainly established during the crack initiation stage. Cutting-induced surface integrity, residual stress, and near-edge microstructure jointly affected local stress concentration and resistance to cyclic damage, thereby controlling fatigue crack initiation and the overall fatigue life.

**Figure 17:**
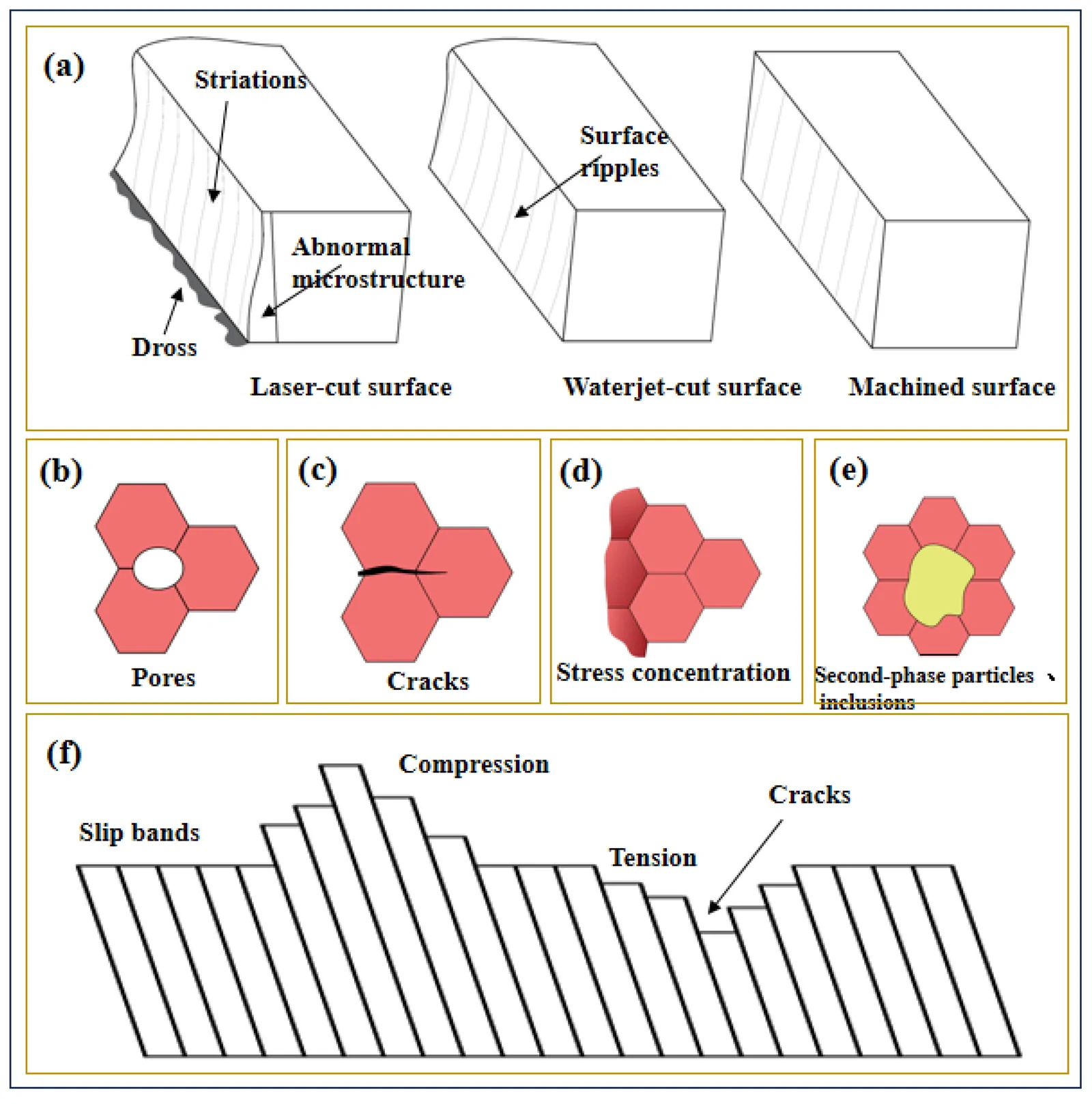
Schematic illustration of the fatigue crack initiation mechanisms: (**a**) crack initiation sites; (**b**–**e**) microdefect-induced mechanisms; (**f**) persistent slip band mechanism.

Figure 17 schematically illustrates the primary fatigue crack initiation mechanisms under different surface conditions. The laser-cut specimens contained relatively deep and sharp periodic surface striations, bottom-edge dross, and abnormal microstructural regions. Under cyclic loading, these defects readily generated local stress concentrations, significantly shortened the crack initiation life, and promoted preferential crack initiation from pre-existing defects, as shown in Figure 17a. The waterjet-cut specimens exhibited relatively good surface quality; however, slight macroscopic waviness was still generated during cutting, while abrasive impact produced microscopic pits and near-surface work hardening. Consequently, fatigue cracks mainly initiated from the roots of surface waviness or locally damaged regions. The mechanically ground specimens exhibited smoother and more uniform surfaces with fewer macroscopic stress concentration sites and therefore a lower tendency for crack initiation.

When localized defects such as pores, microcracks, or second-phase particles were present, fatigue cracks could initiate from these regions, as illustrated in Figure 17b–e. In the absence of pronounced surface defects, crack initiation was mainly governed by the persistent slip band mechanism. Under cyclic loading, localized plastic slip developed within near-surface grains and progressively evolved into fatigue cracks, as shown in Figure 17f.

Fatigue cracks generally preferentially initiate at regions of local stress concentration associated with surface defects. Therefore, improving surface quality and reducing the stress concentration factor, *K_t_*, are beneficial for enhancing the fatigue performance of the material. The value of *K_t_* is jointly influenced by material properties, specimen geometry, loading mode, and surface morphology. In the present study, all specimens had the same macroscopic dimensions and were subjected to identical loading conditions, while the differences in tensile properties were relatively limited. Therefore, the observed differences in fatigue behavior were mainly attributed to variations in surface roughness and surface integrity induced by the different cutting processes. To quantitatively evaluate the effect of surface roughness on the fatigue performance of 5083-H111 aluminum alloy, the relationship between *K_t_* and surface roughness parameters proposed by Arola et al. [26] was employed:

(4) $$K_{t}=1+n\sqrt{\lambda \left(\frac{R_{a}}{\rho }\right)\left(\frac{R_{y}}{R_{z}}\right)}$$

where *n* is the stress-state parameter, with *n* = 1 for shear loading and *n* = 2 for tensile loading; *λ* is the ratio of notch spacing to surface-profile depth and is generally taken as *λ*; *ρ* is the radius of curvature at the notch root; *Ra* is the arithmetic mean roughness; *Ry* is the maximum peak-to-valley height of the surface profile; and *Rz* is the ten-point mean roughness. The notation *Ry* follows that used by Arola and Williams [26] and is also referred to as Rmax in some roughness standards.

In addition to surface roughness, the residual stress field can also modify the local near-surface stress state and thereby influence fatigue crack initiation and short-crack propagation. In general, a higher surface residual compressive stress provides a stronger counteracting effect against the externally applied cyclic tensile stress, reducing the effective tensile stress experienced at the material surface and consequently delaying fatigue crack initiation. Therefore, residual compressive stress is generally beneficial to fatigue performance, whereas a reduction in compressive residual stress or the presence of residual tensile stress may promote crack initiation and propagation.

The near-surface microstructure is also an important factor affecting fatigue performance. The 5083-H111 aluminum alloy used in this study was in the rolled condition, and the high dislocation density and deformation substructures retained in the surface layer could, to some extent, restrict localized plastic deformation, thereby delaying fatigue crack initiation and early-stage crack propagation. Waterjet cutting is essentially a cold-working process, and high-velocity abrasive impact produces a work-hardened surface layer and further increases the near-surface dislocation density, which is beneficial for enhancing resistance to fatigue crack initiation. In contrast, the relatively high heat input generated during laser cutting promotes recovery, recrystallization, and even localized remelting near the cut edge, thereby weakening or eliminating the dislocation-strengthening effect inherited from the original rolled microstructure. The resulting fine equiaxed-grain structure consequently reduces the resistance of the surface layer to cyclic damage.

To quantitatively characterize the combined effects of surface roughness, residual stress, and near-surface microstructure, Wang et al. [27] introduced a fatigue risk factor (*Rf*) to evaluate the susceptibility of metallic materials to fatigue cracking under cyclic loading. A higher *Rf* value indicates a greater risk of fatigue crack initiation and failure. The fatigue risk factor is expressed as follows:

(5) $$R_{f}=\frac{K_{t}\sigma_{e}+\sigma_{r}}{\sigma_{y}}$$

where *σ_e_* is the applied stress, *σ_r_* is the residual stress, *σ_y_* is the yield strength, and *K_t_* is the stress concentration factor.

The fatigue risk factor (*Rf*) was calculated using Equation (5) with an applied stress of 150 MPa. The parameters include the stress concentration factor (*K_t_*), residual stress (*σr*), yield strength (*σy*), and applied stress (*σe*). Compressive residual stress was assigned a negative value. Based on the parameters in Table 3, the calculated Rf values were 0.16, 0.36, and 1.32 for mechanical grinding, waterjet cutting, and laser cutting, respectively. The higher Rf of the laser-cut specimens indicates a greater susceptibility to fatigue crack initiation. A sensitivity analysis was performed by varying each input parameter by ±10% while keeping the others constant.

## 5. Conclusions

(1) The effects of different cutting processes on kerf-surface morphology and near-edge microstructure were revealed. The mechanically ground specimen and waterjet-cut specimens retained the typical features of the rolled microstructure near the kerf edge, characterized by a high dislocation density, strong crystallographic texture, and high residual compressive stress. For the waterjet-cut specimens, the high-velocity impact-induced plastic deformation further refined the grains and intensified local stress concentration, resulting in pronounced work hardening on the cut surface. In contrast, the high heat input during laser cutting induced localized remelting, transforming the near-edge microstructure into fine equiaxed grains with a more randomized texture, while markedly reducing the dislocation density and residual stress.
(2) The differences in mechanical properties and fracture mechanisms among specimens produced by different cutting processes were investigated. Owing to work hardening, the hardness near the waterjet-cut edge increased to approximately 100 HV, whereas softening occurred near the laser-cut edge, with the hardness decreasing to approximately 50–60 HV. Compared with the mechanically ground specimens, the waterjet-cut specimens exhibited a slightly lower ultimate tensile strength of 332.1 MPa, while the tensile strength of the laser-cut specimens decreased to 295.8 MPa. After removal of the bottom-edge dross, the local sharp stress-concentration sources were eliminated and the tensile strength slightly increased, although the improvement was limited. Fractographic analysis showed that the mechanically ground specimen and waterjet-cut specimens were dominated by ductile fracture, whereas the laser-cut specimens exhibited a mixed brittle–ductile fracture mode.
(3) The influence of different cutting processes on the high-cycle fatigue performance of the aluminum alloy was systematically investigated. At 1 × 10^7^ cycles, the fatigue strength of the laser-cut specimens without dross removal was 60.19 MPa, which increased to 67.64 MPa after dross removal but remained lower than that of the waterjet-cut specimens (99.21 MPa). Dross removal partially alleviated local stress concentration and improved fatigue performance; however, the inherent surface striations generated by laser cutting and the degradation of the heat-affected-zone microstructure remained the primary causes of the reduced fatigue strength. Fractographic analysis showed that fatigue cracks in the laser-cut specimens initiated mainly at the dross roots and microscopic defects associated with periodic surface striations. In the waterjet-cut specimens, cracks originated from local stress-concentration regions associated with geometric notches formed by cutting waviness, whereas fatigue cracks in the mechanically ground specimens were mainly induced by fine surface scratches.
(4) By introducing the fatigue risk factor (*R_f_*), the cracking tendency of the material under cyclic loading was quantitatively evaluated for different cutting processes, and the corresponding fatigue crack-initiation and failure mechanisms were clarified. Owing to the high surface roughness, severe stress concentration, and softening of the abnormal microstructural region near the kerf edge, the laser-cut specimens exhibited a markedly increased driving force for crack initiation. The fatigue risk factor of the laser-cut specimens (*R_f_* = 1.32) was substantially higher than those of the waterjet-cut specimens (*R_f_* = 0.36) and mechanically ground specimens (*R_f_* = 0.16), corresponding to the lowest fatigue life.

## Figures and Tables

**Table 1:** Chemical composition of 5083-H111 aluminum alloy (wt.%).

Element	Cu	Fe	Mn	Mg	Zn	Cr	Ti	Pb/Bi	Al
Content	0.1	0.4	0.4–1.0	4.0–4.9	0.1	0.05–0.25	0.15	-	Balance

**Table 2:** Physical properties of 5083-H111 aluminum alloy.

Physical Properties	Melting Point	Density	Electrical Conductivity	Electrical Resistivity
5083-H111 aluminum alloy	570–640 °C	2.66 g/cm^3^	29–34% IACS	0.059 Ω/m

**Table 3:** Key parameters affecting the fatigue performance of 5083-H111 aluminum alloy.

	*Ra* (μm)	*Ry* (μm)	*Rz* (μm)	*ρ* (μm)	*K_t_*	*σ_r_* (MPa)	*σ_y_* (MPa)	*R_f_*
Mechanical grinding	0.8	1.21	4.25	72	1.11	−130	231.5	0.16
Waterjet cutting	6.5	23.5	50.83	26	1.68	−170	225.4	0.36
Laser cutting	26.2	47.93	260.21	8	2.55	−90	182.6	1.32

## Data Availability

The raw data supporting the conclusions of this article will be made available by the authors on request.
